# Impact of sex differences on thrombin-induced hydrocephalus and white matter injury: the role of neutrophils

**DOI:** 10.1186/s12987-021-00273-0

**Published:** 2021-08-16

**Authors:** Kang Peng, Sravanthi Koduri, Fan Xia, Feng Gao, Ya Hua, Richard F. Keep, Guohua Xi

**Affiliations:** 1 Department of Neurosurgery, University of Michigan, R5018 Biomedical Science Research Building, 109 Zina Pitcher Place, Ann Arbor, MI 48109-2200 USA; 2 Department of Neurosurgery, Xiangya Hospital, Central South University, Changsha, China

**Keywords:** Thrombin, Male, Female, Neutrophils, Hydrocephalus, White matter injury

## Abstract

**Background:**

Thrombin has been implicated in playing a role in hydrocephalus development following intraventricular hemorrhage (IVH). However, the mechanisms underlying the sex differences to the detrimental effects of thrombin post-IVH remain elusive.

**Method:**

Three-month old male and female Sprague-Dawley rats underwent unilateral intracerebroventricular (ICV) injections of 3U or 5U thrombin, or saline, to examine differences in thrombin-induced hydrocephalus and white matter injury. Mortality, and lateral ventricle volume and white matter injury were measured on magnetic resonance imaging evaluation at 24 h post-injection. In addition, male rats were pretreated with 17-β estradiol (E2, 5 mg/kg) or vehicle at 24 and 2 h prior to ICV injection of 3U thrombin. All rats were euthanized at 24 h post-injection for histology and immunohistochemistry.

**Results:**

ICV injection of 5U thrombin caused 100 and 0% mortality in female and male rats, respectively. 3U of thrombin resulted in significant ventricular dilation and white matter damage at 24 h in both male and female rats, but both were worse in females (p < 0.05). Furthermore, neutrophil infiltration into choroid plexus and periventricular white matter was enhanced in female rats and may play a critical role in the sex difference in brain injury. Pre-treating male rats with E2, increased thrombin (3U)-induced hydrocephalus, periventricular white matter injury and neutrophil infiltration into the choroid plexus and white matter.

**Conclusions:**

ICV thrombin injection induced more severe ventricular dilation and white matter damage in female rats compared to males. Estrogen appears to contribute to this difference which may involve greater neutrophil infiltration in females. Understanding sex differences in thrombin-induced brain injury may shed light on future interventions for hemorrhagic stroke.

## Background

Intraventricular hemorrhage (IVH) is a common complication of intracerebral hemorrhage (ICH) and subarachnoid hemorrhage (SAH) in adults, and for germinal matrix hemorrhage in newborns [1–3]. Hydrocephalus occurs frequently after IVH and it is correlated with higher hemorrhage-associated mortality [4, 5]. Ventricular zone disruption and ependymal denudation or discontinuities have been observed in human neonates with IVH or communicating hydrocephalus [6, 7]. Furthermore, accumulating evidence implicates hydrocephalus as a potential cause of axonal and myelin damage in the periventricular white matter (WM), which may contribute to poor behavioral outcomes after IVH [8, 9].

Thrombin is an essential component of the coagulation cascade and is produced in the brain immediately after a hemorrhagic event to induce hemostasis [10]. Thrombin has been shown to participate in brain injury after both ICH and cerebral ischemia [11–13]. Our previous studies have also demonstrated that thrombin is involved in hydrocephalus development after IVH, and intracerebroventricular (ICV) injection of thrombin can cause severe hydrocephalus and induce neuroinflammation within the choroid plexus [14, 15].

Sex is an influential factor impacting the severity of brain injury following hemorrhagic stroke. Several studies have reported a higher incidence of mortality following IVH in premature male infants, compared to female infants [16, 17]. Additionally, our previous studies have shown that female mice have reduced cerebral edema and improved behavioral recovery following ICH [18]. Furthermore, estrogen has been found to reduce iron-mediated intracerebral edema and neuronal death [19]. These findings suggest that the female sex may be protected after IVH and ICH. However, previous studies have shown that women had higher morbidity and worse brain injury after suffering SAH [20]. In addition, some clinical investigations indicate that being female is an independent factor for higher incidence of hydrocephalus after SAH [21, 22]. The current study investigates whether there are sex differences in thrombin-induced hydrocephalus and periventricular WM injury, and further elucidates the underlying mechanisms.

## Methods

### Animal preparation and intraventricular injection

All animal protocols were approved by the University of Michigan Institutional Animal Care and Use Committee. A total of forty-eight 3-month-old Sprague-Dawley rats (Charles River Laboratories, Portage, MI), including 30 male and 18 female rats, were used in this study. Animals were anesthetized with pentobarbital (50 mg/kg intraperitoneally) and core body temperature was maintained at 37.5 °C during intraventricular injection and recovery from anesthesia. Rats received an ICV infusion of saline or thrombin into the right lateral ventricle (coordinates: 0.6 mm posterior, 4.5 mm ventral, and 1.6 mm lateral to the bregma).

### Experimental groups

This study included 3 parts: (1) male and female rats (n = 6 for each) underwent an unilateral ICV injection of 5 units (5U) rat thrombin (Sigma-Aldrich) dissolved in saline (50 µl); (2) rats of both sexes received an unilateral ICV injection of 3 units (3U) thrombin dissolved in saline or saline alone (50 µl) for a total of four groups (n = 6 for each group); (3) male rats were pretreated with 17-β estradiol (E2; 5 mg/kg dissolved into saline with 1% gelatin, subcutaneous) or vehicle (saline with 1% gelatin) at 24 and 2 h prior to the ICV injection of 3U thrombin (n = 6 for each group). All injections were performed over 10 min. Following MRI at 24 h post-ICV injection, rats were euthanized for immunohistochemistry analysis.

### MRI scanning and measurement

All rats underwent MRI at 24 h after saline or thrombin ICV injections. MRI was performed in a 7.0-T Varian MR scanner (Varian Inc.) with a T2 fast spin-echo sequence using a field of view of 35mm×35 mm and obtaining a total of 25 coronal slices. Ventricle volume and WM damage area were measured as described previously [23–25]. Briefly, bilateral lateral ventricles as well as WM T2-hyperintensity area were outlined and quantified all slices using Image J (National Institutes of Health, USA) by a blinded observer.

### Hematoxylin & eosin and immunohistochemistry staining

Rats were euthanized with pentobarbital (100 mg/kg, intraperitoneally) and perfused with 4% paraformaldehyde. Brains were removed and placed in 4% paraformaldehyde for 24 h at 4 °C prior to immersion in 30% sucrose for 48–72 h at 4 °C. Brains were then embedded in optimal cutting temperature compound (Sakura Finetek USA) and sectioned on a cryostat (18-µm-thick sections). Hematoxylin and eosin (H&E) staining was used to measure the ventricular wall damage as well as neutrophil infiltration (by morphology). Immunohistochemical studies were performed using the avidin-biotin complex technique as previously described [26]. The primary antibody was rabbit anti-myeloperoxidase (MPO; 1:200; Invitrogen) and the secondary antibody was goat anti-rabbit (1:500; Invitrogen). Negative controls omitted the primary antibody. Hematoxylin was used for contrast staining.

### Cell counting and immunoactivity determination

To assess the severity of ventricular wall damage and avoid the area of direct damage due to the ICV injection, the temporal horns of the lateral ventricles (coronal brain section between − 3.8 and −4.3 mm posterior to bregma) were chosen and the ratio of disrupted ependymal surface to total ventricular wall length within one slice was calculated as a percentage. For MPO positive cells counts, three random high-power images (×40 magnification) were obtained of the choroid plexus (based on the coronal brain sections between − 0.4 and −0.9 mm posterior to bregma) and periventricular WM from bilateral lateral ventricles, and the number of MPO positive cells is represented as cells per square millimeter. All analyses were completed with ImageJ by a blinded observer.

### Statistical analysis

Values are given as means ± SD. Student *t*-test and two-way ANOVA were used for analysis. Differences were considered significant at *p* < 0.05.

## Results

Intracerebroventricular injection of 5U of rat thrombin caused 100% mortality in female rats (6/6) but there were no deaths in male rats (0/6). When 3U thrombin or saline were injected ICV, there were no deaths in either female or male rats. Our previous study showed that ICV injection of thrombin (3U) resulted in ventricular enlargement as determined by MRI [14]. Therefore, we used 3U thrombin in the remainder of this study.

### Intracerebroventricular injection of thrombin induced ventricular dilation and white matter damage in male rats.

**Fig. 1:**
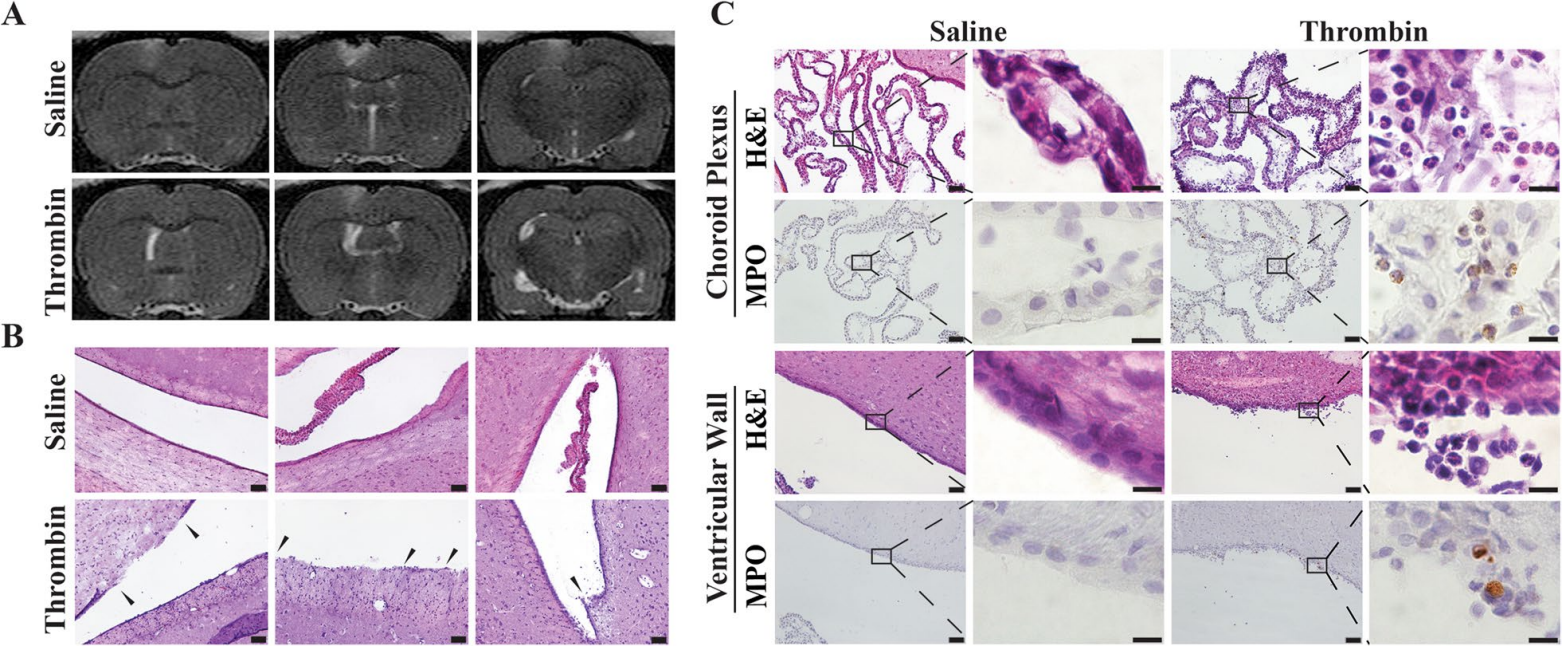
Intracerebroventricular injection of thrombin induced severe ventricular dilation, ventricular wall damage, and neutrophil infiltration in male rats. **A** T2 weighted MRI showing ventricular volume at 24 h after ICV injection of 50 µl of saline or thrombin (3U) in male rats. The bottom left image of this panel has been published previously [15]. **B** Representative images of H&E staining showing ependymal denudation and rupture (arrows) at 24 h in the thrombin (3U) but not the saline group. Scale bar = 50 μm. **C** Representative H&E and myeloperoxidase (MPO) staining of the choroid plexus and ventricle wall 24 h after thrombin or saline injection. Note the neutrophil infiltration into the choroid plexus and the ventricular wall damage in the thrombin injection group. Lower magnification, scale bar = 50 μm; higher magnification, scale bar = 10 μm

Intracerebroventricular thrombin (3U) increased lateral ventricle volume in male rats compared to saline injection at 24 h post-injection (Fig. 1A). Additionally, compared to the saline group, greater ventricular wall damage as determined based on the disruption of the ependymal layer, sparse WM and cellular shedding, was observed in male rats with ICV thrombin (Fig. 1B).

Neuroinflammation within the choroid plexus may contribute to the occurrence of hydrocephalus. Our previous study revealed that ICV thrombin injection mimics the choroid plexus inflammation that was observed after subarachnoid hemorrhage [15]. In the current study, neuroinflammation within the choroid plexus was detected at 24 h after ICV thrombin injection by H&E staining with marked polymorphonuclear neutrophil infiltration in male rats. MPO staining confirmed that ICV thrombin injection induced choroid plexus neutrophil infiltration. Even within the ventricular wall, we also found there were numerous neutrophils infiltration within the ependymal cells layer, which may contribute to the ventricular wall damage (Fig. 1C).

**Fig. 2:**
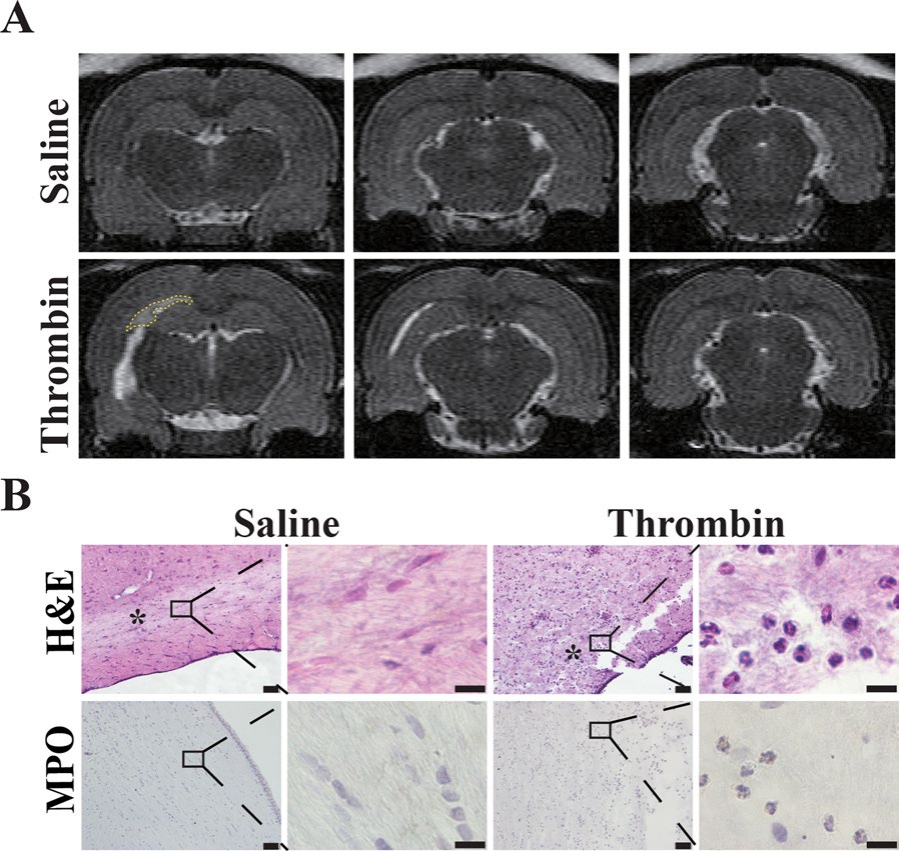
Intracerebroventricular injection of thrombin induced white matter damage and neutrophil infiltration in male rats. **A** T2 weighted MRI showing white matter hyperintensity (outline) at 24 h after ICV injection of thrombin (3U) injection but not saline injection in male rats. **B** Representative H&E and MPO staining images showing neutrophil infiltration into the white matter injury area at 24 h in the thrombin injection group. * indicates white matter area. Lower magnification, scale bar = 50 μm; higher magnification, scale bar = 10 μm

Periventricular T2-hyperintensity, a marker of WM injury, has been shown to be induced in rats after ICV thrombin injection, but not saline injection [27]. In the current experiments, periventricular T2-hyperintensity area was also found to be larger in male rats who underwent thrombin injection than with saline injection at 24 h post-injection (Fig. 2A). To gain further understanding of the potential mechanism of thrombin-induced WM damage, MPO staining was performed and showed marked neutrophil infiltration into the periventricular WM injury region following ICV injection of thrombin but not saline in male rats (Fig. 2B). Based on the above data, neutrophils may play a role in thrombin-induced periventricular WM injury.

### Intracerebroventricular injection of thrombin induced more severe ventricle dilation and white matter damage in female rats compared to males

**Fig. 3:**
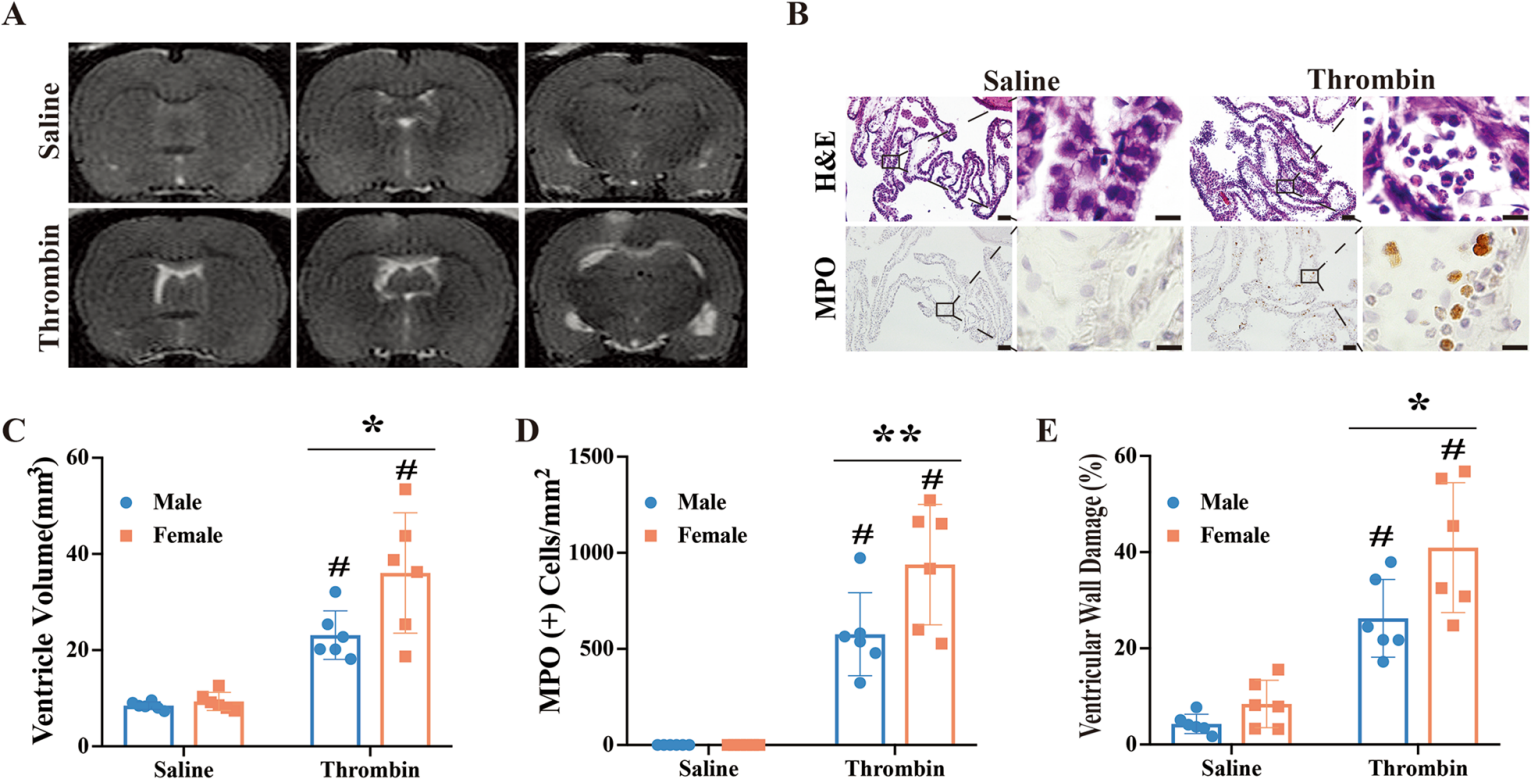
Intracerebroventricular injection of thrombin induced greater ventricular dilation, worse ventricular wall damage, and more neutrophil infiltration into choroid plexus in female rats compared to males. **A** T2 weighted MRI showing ventricular volume at 24 h after ICV injection of 50 µl of saline or thrombin (3U) in female rats. **B** Representative H&E and myeloperoxidase (MPO) staining images showing neutrophil infiltration into the choroid plexus after ICV injection of thrombin but not saline. Lower magnification, scale bar = 50 μm; higher magnification, scale bar = 10 μm. **C** Quantification of ventricular dilation in the male and female rats. **D** Quantification of MPO positive cell infiltration into the choroid plexus in male and female groups. **E** Quantification of ventricular wall damage in the male and female groups. Values are expressed as the means ± SD, n = 6 in each group, **#** p < 0.01 vs. saline, *p < 0.05, **p < 0.01

To investigate differences in injury due to sex, 3U of thrombin was injected ICV in female rats. Similar to male rats, ICV thrombin induced ventricular dilation in female rats at 24 h (36.1 ± 12.5 mm^3^) compared to saline injection (9.3 ± 1.9 mm^3^, p < 0.01; Fig. 3A and C). Furthermore, ventricular volume after ICV thrombin was significantly higher in female rats compared to males (36.1 ± 12.5 vs. 23.1 ± 5.1 mm^3^ in male group, p < 0.05; Fig. 3A and C). There was no significant difference in ventricular volume between female and male saline-injected groups (9.3 ± 1.9 vs. 8.5 ± 0.8 mm^3^, respectively; p > 0.05, Fig. 3A and C).

Interestingly, there was a very robust increase in polymorphonuclear neutrophil infiltration within the choroid plexus at 24 h after ICV thrombin injection in female rats (Fig. 3B). MPO staining confirmed that ICV thrombin-induced neutrophil infiltration into the choroid plexus of female rats (938 ± 313 vs. 0 per mm^2^ in saline group, p < 0.01; Fig. 3B and D). When quantified, there was almost a 2-fold higher density of neutrophils within the choroid plexus of female rats compared to male rats at 24 h after thrombin injection (938 ± 313 vs. 576 ± 216 per mm^2^ in male rats, p < 0.01; Fig. 3D). Similarly, ICV thrombin caused marked ventricular wall damage in female rats (40.9 ± 8.5 % vs. 8.4 ± 4.9% in saline group, p < 0.01; Fig. 3E) and this damage was greater than that observed in male rats at 24 h after thrombin injection (40.9 ± 8.5% vs. 26.3 ± 8.1% in male group, p < 0.05; Fig. 3E).

**Fig. 4:**
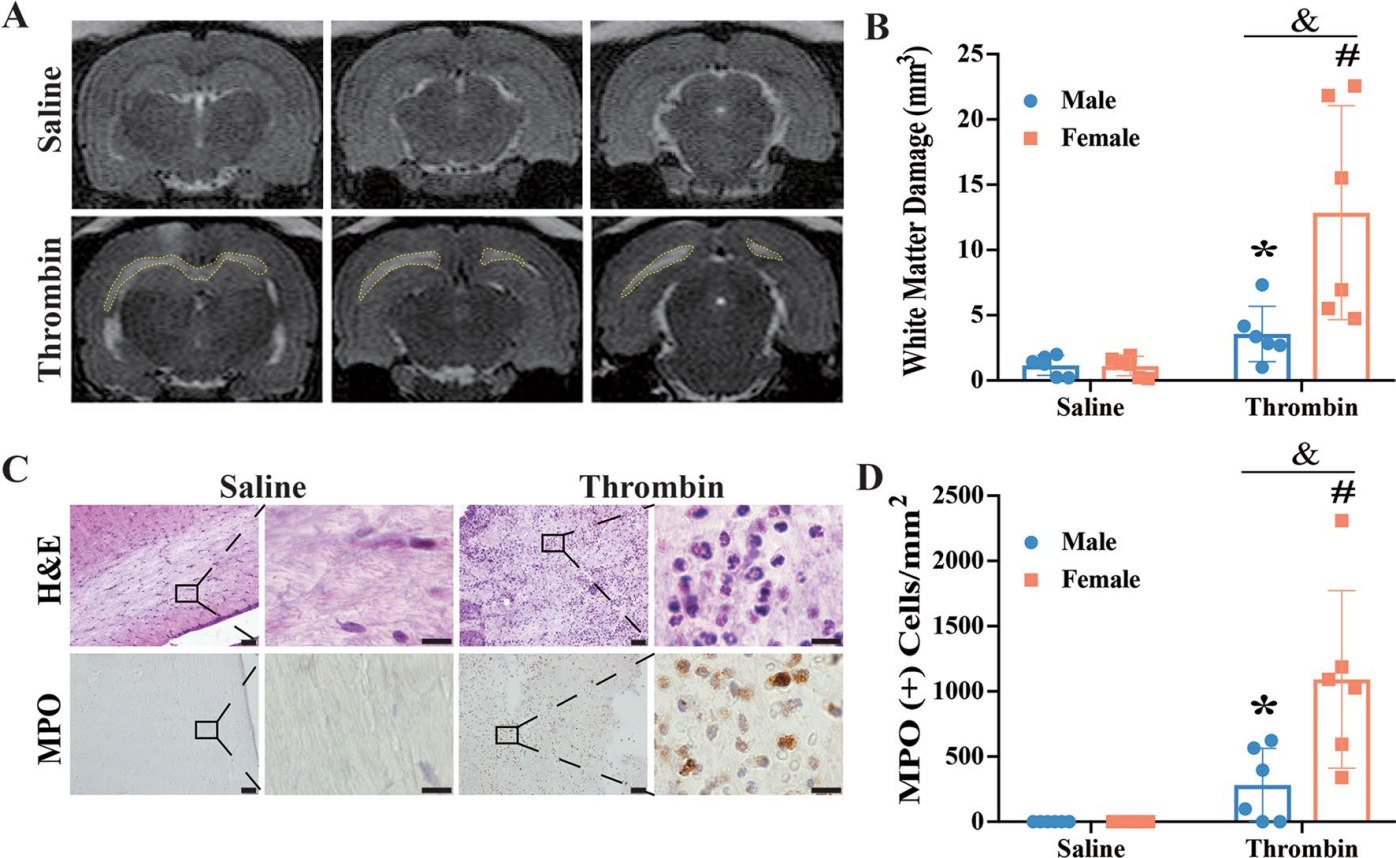
Intracerebroventricular injection of thrombin induces worse white matter damage and more white matter neutrophil infiltration in female compared to male rats. **A** Representative T2 weighted MRI showing white matter hyperintensity (outline) at 24 h after ICV injection of thrombin (3U) but not saline injection in female rats. **B** Quantification of white matter injury in male and female rats 24 h after ICV injection of 3U thrombin or saline. **C** Representative H&E and MPO staining images showing neutrophil infiltration into the white matter injury region at 24 h after ICV injection of thrombin (3U) but not saline injection in female rats. **D** Quantification of MPO positive cells within the white matter injury region in male and female rats 24 h after ICV injection of 3U thrombin or saline. Values are expressed as the means ± SD, n = 6 in each group; *p < 0.05, #p < 0.01 vs. saline. ^&^p < 0.05. Lower magnification, scale bar = 50 μm; higher magnification, scale bar = 10 μm

Compared to the saline group, female rats injected ICV with thrombin had a much larger periventricular T2-hyperintensity lesion at 24 h (12.8 ± 8.2 vs. 1.1 ± 0.7 mm^3^ in saline group, p < 0.01; Fig. 4A). The female rat thrombin injection group also had more WM injury than the male rat thrombin injection group (12.8 ± 8.2 vs. 3.6 ± 2.1 mm^3^ in male rats, p < 0.05, Fig. 4B). Similar to male rats, female rats injected with thrombin ICV showed a robust increase in neutrophils within the region of WM injury when compared to saline injected rats (Fig. 4C). The number of MPO positive cells was calculated within the WM injury region and were drastically higher following thrombin injection (1092 ± 680 vs. 0 per mm^2^ in saline group, p < 0.01; Fig. 4D). In female rats, the number of neutrophils within the WM injury after ICV thrombin was significantly higher than in male rats (1092 ± 680 vs. 281 ± 284 per mm^2^ in male group, p < 0.05; Fig. 4D). Altogether, these data indicate that ICV thrombin induced more severe brain damage in female rats compared to male rats and that neutrophils may contribute to this difference. It should be noted that thrombin-induced ventricular injury was bilateral.

### 17-β estradiol pre-treatment in male rats exacerbates the ventricular dilation, WM injury and neutrophil infiltration induced by ICV injection of thrombin

**Fig. 5:**
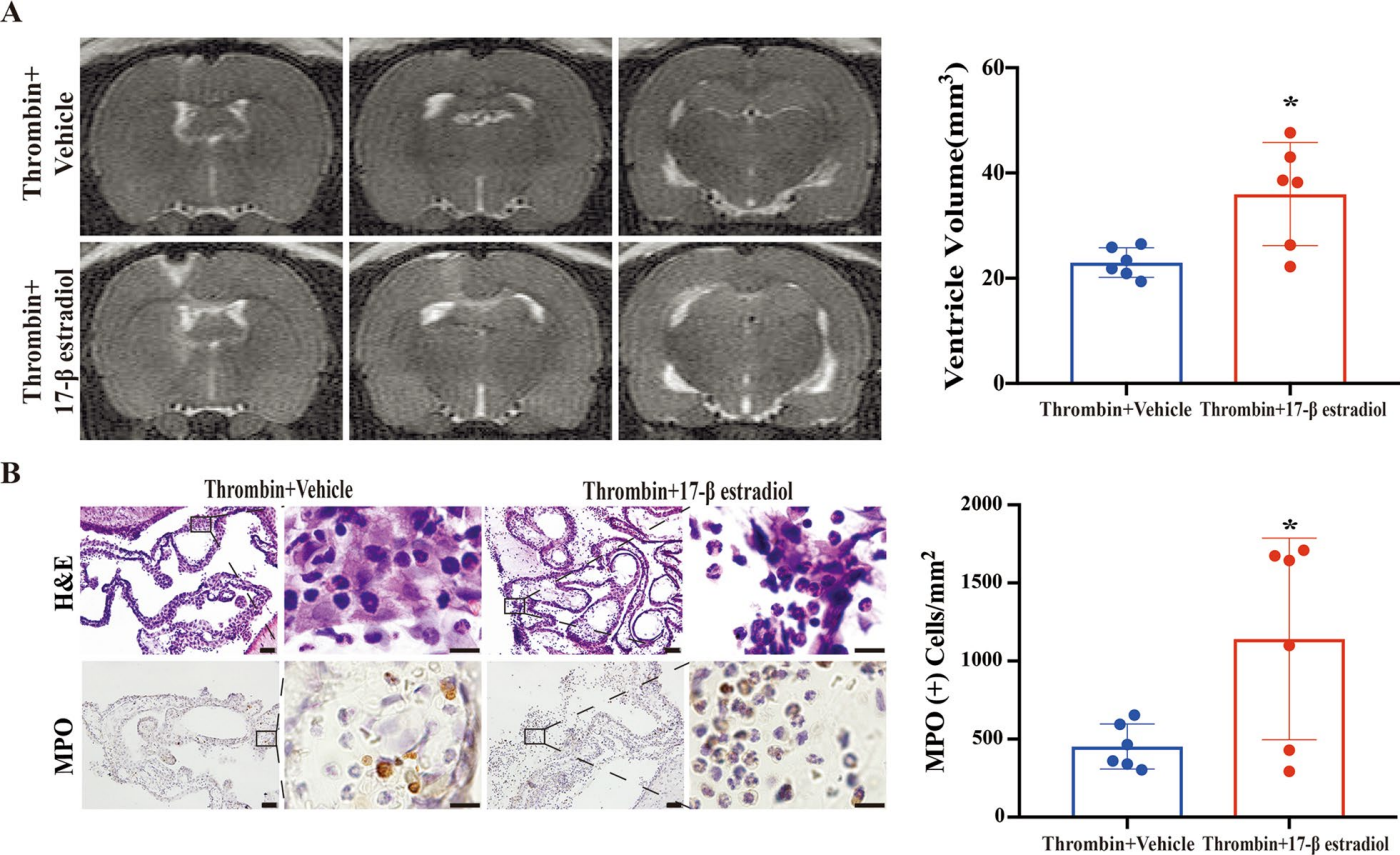
Pre-treatment with 17-β estradiol in male rats leads to greater ventricular dilation and more neutrophil infiltration into choroid plexus after ICV thrombin injection. **A** T2 weighted MRI showing ventricular volume 24 h after ICV thrombin injection in male rats pretreated with 17-β estradiol or vehicle. Quantification of ventricular volume for each group is shown in the bar chart. **B** Representative H&E and MPO staining images showing neutrophil infiltration into the choroid plexus 24 h after ICV injection of thrombin in male rats pretreated with 17-β estradiol or vehicle. Quantification of the number of MPO positive cells in each group is shown in the bar chart. Values are expressed as the means ± SD, n = 6, *p < 0.05 vs. vehicle treatment group. Lower magnification, scale bar = 50 μm; higher magnification, scale bar = 10 μm

To further understand the potential mechanism underlying the sex differences noted above, male rats were treated with 17-β estradiol via subcutaneous injection at 24 and 2 h before ICV thrombin injection. Thrombin injection induced greater ventricular enlargement in the estradiol pre-treatment group at 24 h post-injection compared to the vehicle pre-treatment group (36.0 ± 9.8 vs. 23.4 ± 3.5 mm^3^ in vehicle, p < 0.05; Fig. 5A).

This increase in ventricular volume was accompanied by a concomitant increase in neutrophil infiltration into choroid plexus in both lateral ventricles in the estradiol pre-treatment group compared to the vehicle pre-treatment group (Fig. 5B). Myeloperoxidase staining, which was consistent with neutrophil identification on H&E staining, showed a greater than 2-fold increase in MPO (+) cells in the estradiol pre-treatment group compared to the vehicle group (1142 ± 646 vs. 452 ± 144 per mm^2^ in vehicle, p < 0.05; Fig. 5B).

**Fig. 6:**
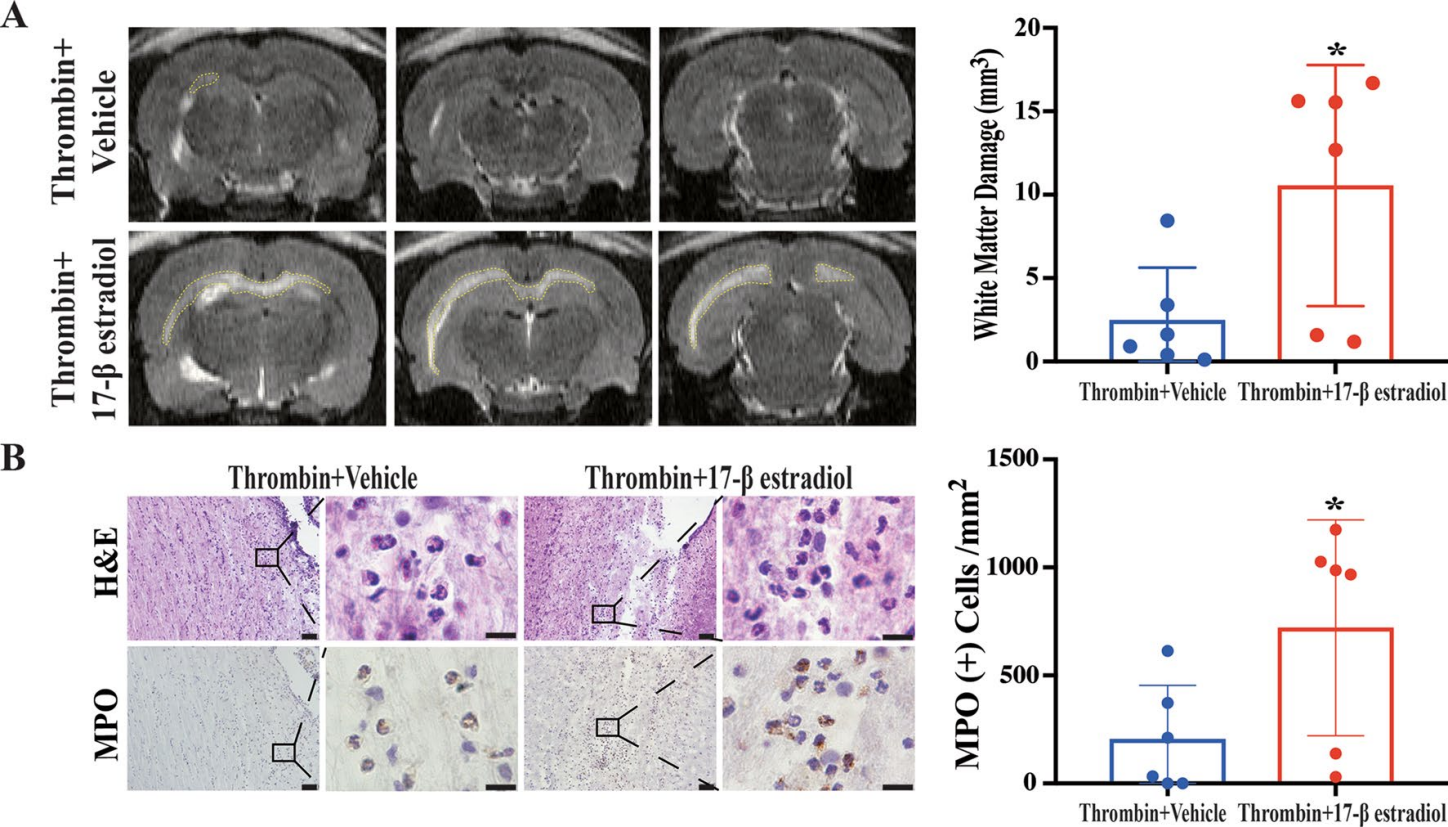
Pre-treatment with 17-β estradiol in male rats leads to increased white matter damage and more neutrophil infiltration after ICV injection of thrombin. **A** T2 weighted MRI showing white matter hyperintensity (outline) at 24 h after thrombin intraventricular injection in male rats pretreated with 17-β estradiol or vehicle. Quantification of the white matter damage in each group is shown in the bar chart. **B** Representative H&E and MPO staining images showing more neutrophil infiltration into the white matter injury region 24 h post thrombin-injection in male rats pretreated with 17-β estradiol or vehicle. Quantification of the number of MPO positive cells in each group is shown in the bar chart. Values are expressed as the means ± SD, n = 6 in each group; *p < 0.05 vs. male vehicle treatment group. Lower magnification, scale bar = 50 μm; higher magnification, scale bar = 10 μm

In addition, 17-β estradiol pre-treatment in male rats also resulted in greater thrombin-induced WM damage on MRI compared to the vehicle group (10.5 ± 7.2 vs. 2.5 ± 3.2 mm^3^ in vehicle, p < 0.05; Fig. 6A). This increased damage with estradiol pre-treatment was accompanied by an increase in the number of polymorphonuclear neutrophils after ICV thrombin in the estradiol pre-treated group (Fig. 6B). This was confirmed by an increased number of MPO positive cells in the WM injury region in 17-β estradiol treated male rats (721 ± 500 vs. 205 ± 249 per mm^2^ in vehicle, p < 0.05; Fig. 6B). These data indicate that 17-β estradiol treatment worsens WM damage in the male ICV thrombin injection model.

### Neutrophil infiltration correlates with ventricular dilation and WM injury induced by unilateral ICV injection of thrombin

**Fig. 7:**
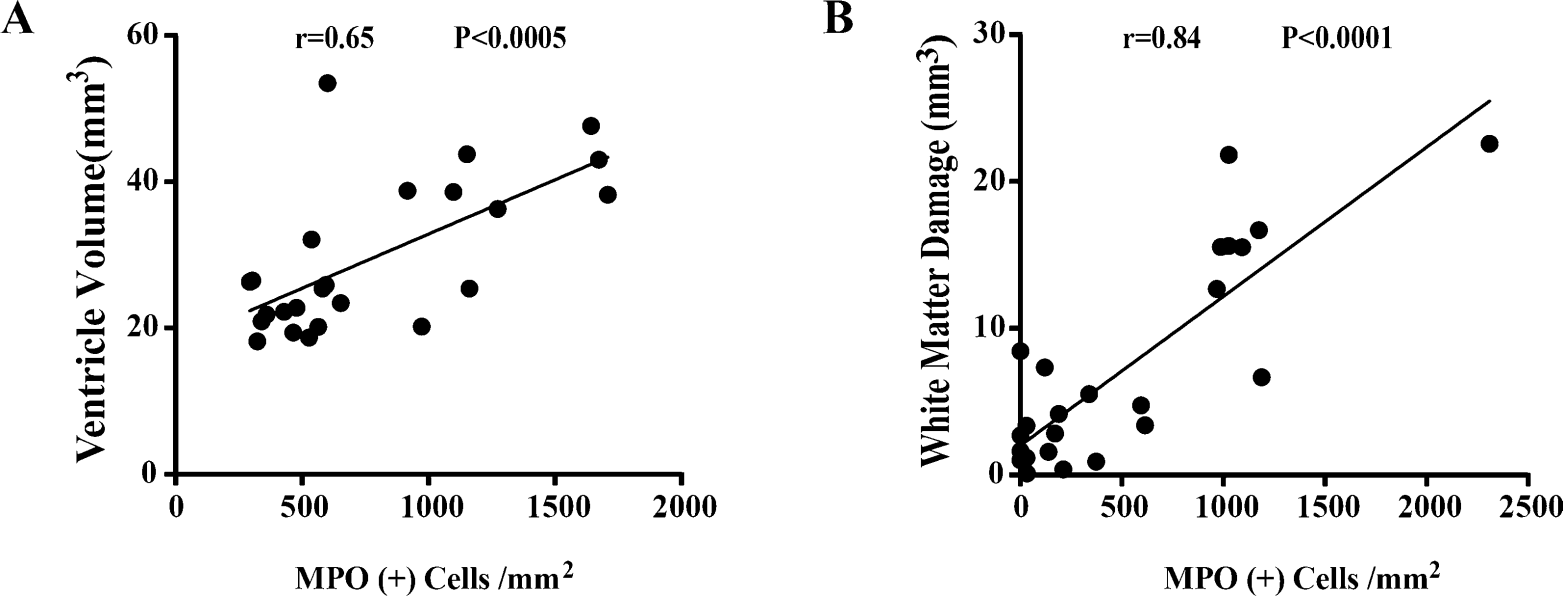
Neutrophil infiltration correlates with the ventricular dilation and WM injury induced by ICV injection of thrombin. **A** Linear regression analysis showing thrombin-induced ventricular dilation correlates with neutrophil infiltration of the choroid plexus. n = 24, p < 0.0005. **B** Linear regression analysis showing thrombin-induced WM injury correlates with neutrophil infiltration of the WM injury area. n = 24, p < 0.0001

To further elucidate whether neutrophil infiltration contributes to ventricular dilation and WM injury, linear regression analysis was performed in all rats injected with thrombin ICV. There was a positive correlation between ventricular dilation and neutrophil infiltration within the choroid plexus bilaterally (r = 0.65, p < 0.0005, linear regression analysis, Fig. 7A). Furthermore, WM injury also had a positive correlation with neutrophil infiltration within the WM injury region bilaterally (r = 0.84, p < 0.0001, linear regression analysis, Fig. 7B).

Taken together, these data further suggest that neutrophils play a prominent role in the sex differences noted after ICV thrombin injection. This role may be related to hormonal control, particularly with respect to estrogen.

## Discussion

Based on the data presented above, there are three major findings: (1) unilateral ICV thrombin injection causes higher mortality and results in more severe ventricular enlargement and periventricular WM damage in female than male rats; (2) 17-β estradiol pre-treatment aggravates thrombin-induced hydrocephalus and WM injury in male rats; and (3) neutrophils may play a central role in thrombin-induced brain damage as well as in the mechanism leading to sex differences of this pathology. Our prior study showed that thrombin plays a central role in the development of hydrocephalus after IVH, and that ICV injection of thrombin causes significant ventricular dilatation in male rats [14]. This study replicated those results in male rats and extended the finding to female rats and further elucidated the potential mechanisms underlying those pathological changes.

Thrombin is produced immediately after a brain hemorrhage. It is known that 100 µl of whole blood can produce about 26 to 36 units of thrombin [28]. In this study, ICV injection of 5U thrombin in female rats caused 100% mortality but 0% mortality in male rats. Similarly, ICV injection of 3U thrombin induced significantly greater ventricular enlargement, more severe ventricular wall damage, and worse WM injury in female than male rats. Male rats who underwent pretreatment with 17-β estradiol also had greater ventricular enlargement, more severe ventricular wall damage, and worse WM injury than untreated male rats. The degree of ventricular enlargement, however, has been modest compared to the severe ventriculomegaly seen in human IVH. This is thought to be secondary to the localized injection of thrombin into the right lateral ventricle, whereas IVH is diffuse. Also, these injections create a bolus of thrombin at a specific time point rather than the gradual release of thrombin that is observed in IVH. Both of these deviations from human physiology may be causal for this modest ventriculomegaly found in the animal model. Overall, these data indicate that females have worse outcome after ICV thrombin injection with estradiol playing a significant role in the mechanism generating this difference.

Ependymal denudation and discontinuities are the dominant alterations in the damage process associated with IVH and hydrocephalus and may potentially contribute to the development of hydrocephalus [6, 7]. An interesting phenomenon found in the *hyh* mutant mouse, which has congenital moderate communicating hydrocephalus, is the presence of ventral ependymal denudation early in fetal life, even prior to the occurrence of communicating hydrocephalus. This leads to the possibility that ventral ependymal denudation may contribute to the mechanism underlying the development of communicating hydrocephalus [29]. In the current study, ependymal denudation and regions of sparse WM were observed after ICV thrombin injection, consistent with previous reports. A recent study successfully established a preterm IVH model *in vitro* that allows for the exploration of potential pathophysiologic alterations within the ventricular zone [30]. Further study of the effect of thrombin on preterm infants is needed and can be examined in such a model.

The impact of sex on different types of brain injury is complex. There have been multiple studies indicating female animals sustain less severe brain damage after ischemic and hemorrhagic stroke (e.g. [18, 31–33]). Several clinical studies have also demonstrated that, in the human neonatal population, male neonates suffer a higher rate of mortality and secondary hydrocephalus following IVH, especially severe IVH (grades 3 and 4) [16, 17]. In contrast, other studies have shown that there is no significant difference in the development of hydrocephalus after IVH between male and female neonates [34, 35]. However, previous studies have revealed that being female is an independent risk factor associated with hydrocephalus after SAH [21, 22]. In fact, a study from our group indicated that tamoxifen, a selective estrogen receptor modulator, caused hydrocephalus in male rats after ICH [36]. Estrogen administration enhances thrombin generation in the rat model and 17-β estradiol administration has been shown to lead to platelet aggregation and reduce tolerance to thrombin [37, 38]. These studies suggest that estrogen may play a role in promoting the toxic effects of thrombin, in line with the results of the current study. Nonetheless, the exact mechanisms that underlie this sex difference in thrombin-induced hydrocephalus are still unclear, though neutrophils may play an important role.

Neutrophils are a first line of defense of the innate immune system against external microbes [39]. However, several reports have demonstrated that neutrophils can play a deleterious role in intracerebral injury by releasing extracellular traps (NETs). Depletion of neutrophils and subsequent decreased formation of NETs can reduce neuronal death and improve outcomes after ischemic and hemorrhagic stroke [40–42]. Our previous studies have shown that neutrophils may participate in the development of post-hemorrhagic hydrocephalus [43, 44]. However, within the current literature on hydrocephalus, there has been limited investigation of the mechanisms involving neutrophils.

In the current study, a greater than 2-fold increase in neutrophil infiltration into choroid plexus and regions of WM damage was observed at 24 h after ICV thrombin injection in female rats compared to male rats, and male rats pre-treated with 17-β estradiol compared to vehicle, suggesting that neutrophils may play a crucial role in this sex difference. Thrombin, acting as a damage-associated molecular patterns (DAMPs), may enhance the expression of inflammatory adhesion molecules (e.g. VCAM-1, ICAM-1, P-selectin, etc.), which are essential proteins in the process of neutrophil infiltration [45]. 17-β estradiol administration may reduce tolerance to thrombin and therefore, aggravate the resultant toxic effects [37]. Additionally, an *in vitro* study showed that 17-β estradiol could promote neutrophil extracellular trap formation, which has been shown to be a mechanism for causing intracerebral injury [46]. Further investigations of the role of neutrophils in generating the sex differences found in this study are needed.

It should be noted that multiple mechanisms are involved in IVH-, SAH- and ICH-induced brain injury. Thus, for example, neuroinflammation after hemorrhagic stroke involves multiple types of leukocytes and resident microglia. The impact of being female or male, and the role of sex hormones may depend on which injury pathways are activated in particular forms of hemorrhagic stroke.

There are several limitations to this study. Only adult rats were used in this study. Therefore, there is a need for more investigation to fully understand the effect of thrombin on preterm and term infants. Only the effect of estrogen was investigated and there may be other sex hormones involved in creating the observed sex differences. Only two doses of thrombin were given ICV and the form of administration does not fully reflect the human pathophysiology where thrombin is gradually released from an IVH. In addition, only acute experiments within a 24-hour timeframe were completed and therefore, it is unclear whether there may be a recovery back to normal ventricular size and repair of the associated damage or if the damage is permanent. Finally, functional outcomes were not examined, so longer-term studies are needed with behavioral testing to understand the chronic implications of ICV thrombin injection.

## Conclusions

In conclusion, ICV injection of thrombin induced higher mortality, increased ventricular dilation and worsened WM injury in female rats compared to male rats. Those effects were mimicked by pre-treating male rats with estrogen and appear, at least in part, to be related to differences in neutrophil infiltration. These results indicate there may be sex-related targets for reducing IVH-induced brain injury and hydrocephalus.

## Data Availability

The datasets used and/or analyzed in this study are available from the corresponding author (G.X.) upon reasonable request.

## Abbreviations

DAMP: Damage-associated molecular patterns
E2: 17-β estradiol
H&E: Hematoxylin and eosin
ICH: Intracerebral hemorrhage
ICV: Intracerebroventricular
IVH: Intraventricular hemorrhage
MPO: Myeloperoxidase
MRI: Magnetic resonance imaging
NETS: Neutrophil extracellular traps
SAH: Subarachnoid hemorrhage
SD: Standard deviation
U: Units
WM: White matter
