# Correction: impact of sex differences on thrombin‑induced hydrocephalus and white matter injury: the role of neutrophils

**DOI:** 10.1186/s12987-024-00551-7

**Published:** 2024-06-05

**Authors:** Kang Peng, Sravanthi Koduri, Fan Xia, Feng Gao, Ya Hua, Richard F. Keep, Guohua Xi

**Affiliations:** 1 Department of Neurosurgery, University of Michigan, R5018 Biomedical Science Research Building, 109 Zina Pitcher Place, Ann Arbor, MI 48109‑2200 USA; 2 Department of Neurosurgery, Xiangya Hospital, Central South University, Changsha, China

**Correction: fluids barriers CNS 18, 38 (2021)**

10.1186/s12987-021-00273-0

The original publication of this article [1] should have stated that one image in Fig. 1A had been published previously.

This is corrected in the legend below in bold and the original publication has been updated.

Figure 1 Intracerebroventricular injection of thrombin induced severe ventricular dilation, ventricular wall damage, and neutrophil infiltration in male rats. A T2 weighted MRI showing ventricular volume at 24 h after ICV injection of 50 µl of saline or thrombin (3U) in male rats. **The bottom left image of this panel has been published previously** [2]. B Representative images of H&E staining showing ependymal denudation and rupture (arrows) at 24 h in the thrombin (3U) but not the saline group. Scale bar = 50 mm. C Representative H&E and myeloperoxidase (MPO) staining of the choroid plexus and ventricle wall 24 h after thrombin or saline injection. Note the neutrophil infiltration into the choroid plexus and the ventricular wall damage in the thrombin injection group. Lower magnification, scale bar = 50 μm; higher magnification, scale bar = 10 μm.
